# Transcriptome Profiling of Cucumber (*Cucumis sativus* L.) Early Response to *Pseudomonas syringae* pv. *lachrymans*

**DOI:** 10.3390/ijms22084192

**Published:** 2021-04-18

**Authors:** Renata Słomnicka, Helena Olczak-Woltman, Mirosław Sobczak, Grzegorz Bartoszewski

**Affiliations:** 1 Department of Plant Genetics, Breeding and Biotechnology, Institute of Biology, Warsaw University of Life Sciences (SGGW), Nowoursynowska 159, 02-776 Warsaw, Poland; renata_slomnicka@sggw.edu.pl (R.S.); helenaolczak@interia.pl (H.O.-W.); 2 Department of Botany, Institute of Biology, Warsaw University of Life Sciences (SGGW), Nowoursynowska 159, 02-776 Warsaw, Poland; miroslaw_sobczak@sggw.edu.pl

**Keywords:** angular leaf spot disease, defense response, DEGs, transcription factors

## Abstract

Bacterial angular leaf spot disease (ALS) caused by *Pseudomonas syringae* pv. *lachrymans* (*Psl*) is one of the biological factors limiting cucumber open-field production. The goal of this study was to characterize cytological and transcriptomic response of cucumber to this pathogen. Plants of two inbred lines, B10 (susceptible) and Gy14 (resistant), were grown, and leaves were inoculated with highly virulent *Psl* strain 814/98 under growth chamber conditions. Microscopic and transcriptional evaluations were performed at three time points: before, 1 and 3 days post inoculation (dpi). Investigated lines showed distinct response to *Psl*. At 1 dpi bacterial colonies were surrounded by necrotized mesophyll cells. At 3 dpi, in the susceptible B10 line bacteria were in contact with degraded cells, whereas cells next to bacteria in the resistant Gy14 line were plasmolyzed, but apparently still alive and functional. Additionally, the level of H_2_O_2_ production was higher in resistant Gy14 plants than in B10 at both examined time points. In RNA sequencing more than 18,800 transcripts were detected in each sample. As many as 1648 and 2755 differentially expressed genes (DEGs) at 1 dpi as well as 2992 and 3141 DEGs at 3 dpi were identified in B10 and Gy14, respectively. DEGs were characterized in terms of functional categories. Resistant line Gy14 showed massive transcriptomic response to *Psl* at 1 dpi compared to susceptible line B10, while a similar number of DEGs was detected for both lines at 3 dpi. This suggests that dynamic transcriptomic response to the invading pathogen may be related with host resistance. This manuscript provides the first transcriptomic data on cucumber infected with the pathovar *lachrymans* and helps to elucidate resistance mechanism against ALS disease.

## 1. Introduction

Cucumber (*Cucumis sativus* L.) is one of the oldest vegetable crops presently cultivated worldwide in several climatic zones. The total production of cucumbers in 2019 reached almost 88 million tons. China, Russia and Turkey were the major producers [1]. One of the major biotic factors limiting open-field cucumber production is bacterial angular leaf spot (ALS) disease. It was first reported in the United States in 1913, and the causal agent, a Gram-negative bacterium *Pseudomonas syringae* pv. *lachrymans* (*Psl*), was isolated in 1915 by Smith and Bryan [2]. Since then, ALS was reported in many countries worldwide [3]. Its symptoms appear mainly on the leaves, but can also develop on petioles, stems and fruits, leading to significant yield reduction and quality loss [4]. The pathogen colonizes the intercellular spaces of leaf tissues [5] causing vein-limited, water-soaked lesions that turn necrotic. Depending on the cucumber susceptibility and bacteria virulence, a chlorotic halo around necrotic lesions, as well as bacterial ooze on the abaxial leaf side may appear [6,7]. The lesions become an entry point for secondary fungal and bacterial pathogens that colonize leaves and stems [8]. The bacterium can also be transmitted by contaminated seeds [9,10].

There were several studies performed on the genetic background of cucumber ALS resistance [6,11,12]. Recent studies were focused on genetic mapping of ALS resistance. Słomnicka et al. [13], using recombinant inbred lines (RILs), developed from the cross of resistant line Gy14 and susceptible B10, mapped the *psl* locus and identified two quantitative trait loci (QTL), *psl5.1* and *psl5.2,* located next to each other at the short arm of chromosome 5. Wang et al. [14] found a major ALS resistance locus *psl* also at the short arm of chromosome 5 and minor effect loci *psl1.1* and *psl3.1* on chromosomes 1 and 3. Interestingly, the *psl* locus was co-localized with *dm1* and *cla* loci identified as major effect QTL for downy mildew (DM) and anthracnose (AR) resistance. Based on fine mapping of *dm1/psl/cla,* the *CsSGR* gene was proposed as the most likely candidate for this triple-resistance locus. *CsSGR* is cucumber homolog of the *STAYGREEN* gene that encodes chloroplast-located magnesium dechelatase, a key regulator of the chlorophyll degradation pathway and loss-of-susceptibility mutation in *CsSGR* (glutamine-to-arginine substitution in position 108) provides simultaneous resistance to major cucumber diseases: ALS, DM and AR [14]. Recently, an introgression line IL52, derived from *C. sativus* × *C. hystrix* interspecific cross, was identified as highly resistant to ALS, and a single recessive resistance locus *psl-1* was mapped on chromosome 1. The IL52 line can be used as a novel cucumber germplasm for disease resistance breeding in cucumber [15].

A powerful method to study plant response to pathogens is RNA sequencing (RNA-seq). With the advent of RNA-seq, it is now possible to analyze whole-transcriptome changes during plant’s response to pathogen infection. The comparison of transcriptome profiles allows studies of gene networks during pathogen invasion [16]. Transcriptome analysis of well-characterized lines or genotypes is a kind of approach that explores disease resistance mechanisms and reveals the role of various biological pathways. In cucumber, such an approach was applied to study response to viruses, including Cucurbit Chlorotic Yellows Virus (CCYV) and Cucumber Mosaic Virus (CMV) [17,18], oomycete pathogens *Pseudoperonospora cubensis* and *Phytophthora melonis* [19,20,21], and fungal pathogens like *Botrytis cinerea*, *Corynespora cassiicola*, *Fusarium oxysporum* f. sp. *cucumerinum*, *Sphaerotheca fuliginea*, and *Alternaria cucumeriana* [16,22,23,24,25]. RNA-seq was also used to investigate cucumber response to aphid and spider-mite infestations [26,27]. In several plant species, transcriptional changes have been studied to better understand responses of resistant and susceptible plant genotypes to *P. syringae* pathovars, e.g., *Arabidopsis* and *P. syringae* pv. *tomato,* alfalfa and *P. syringae* pv. *syringae,* kiwifruits and *P. syringae* pv. *actinidiae*, and others [28,29,30]. However, to the best of our knowledge, the response of cucumber to bacterial pathogen *P. syringae* pv. *lachrymans* at the transcriptome level has not been studied yet.

The aim of this study was to characterize cytological and transcriptomic changes during ALS disease development to understand more clearly cucumber’s response to *P. syringae* pv. *lachrymans* infection.

## 2. Results

### 2.1. Cucumber Response to P. syringae pv. lachrymans

**Figure 1:**
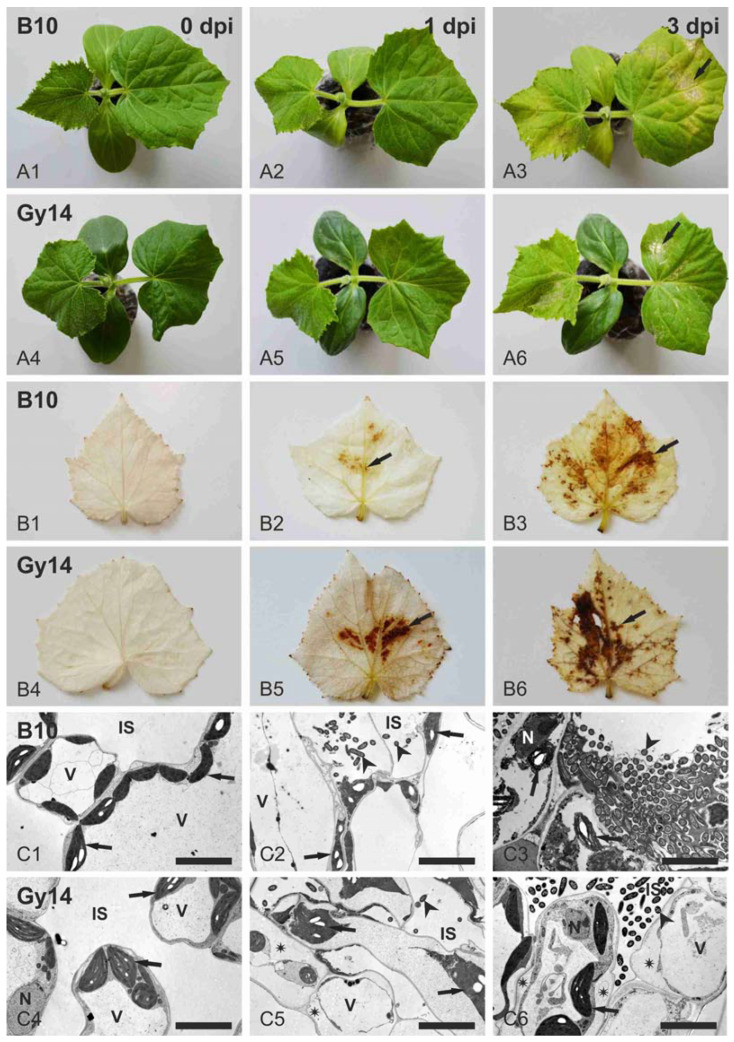
Morphology and ultrastructure of leaves of susceptible B10 and resistant Gy14 cucumber lines upon infection with the *Psl* 814/98 strain (inoculum concentration of 1 × 10^7^ CFU mL^−1^). (**A**) Morphology of inoculated cucumber leaves (arrows indicate selected lesions); (**A1**–**A3**), B10 plants at 0, 1 and 3 dpi, respectively; (**A4**–**A6**), Gy14 plants at 0, 1 and 3 dpi, respectively. (**B**) H_2_O_2_ accumulation in leaves as indicated by staining with DAB (arrows point to selected DAB precipitates); (**B1**–**B3**), B10 plants at 0, 1 and 3 dpi, respectively; (**B4**–**B6**), Gy14 plants at 0, 1 and 3 dpi, respectively. (**C**) Transmission electron microscopy micrographs of leaf mesophyll cells (arrowheads indicate selected bacteria; arrows indicate selected chloroplasts; asterisks indicate regions between plasma membrane and cell wall in plasmolyzed cells); (**C1**–**C3**), B10 plants at 0, 1 and 3 dpi, respectively; (**C4**–**C6**), Gy14 plants at 0, 1 and 3 dpi, respectively. Abbreviations: IS—intercellular space, N—nucleus, V—vacuole. Scale bars, 5 μm (**C**).

Disease symptoms appeared on inoculated leaves of both cucumber lines (i.e., susceptible line B10 and resistant Gy14) after inoculation with the highly virulent *Psl* 814/98 strain using a bacteria concentration of 1 × 10^7^ CFU mL^−1^ (Figure 1A). First symptoms of infection became apparent at 3 dpi, when on B10 leaves chlorosis began to develop (Figure 1A1–A3) causing later necrotic lesions with a large chlorotic halo and angular water-soaked lesions on the abaxial side of infected leaves. At the same time point, the symptoms on leaves of the resistant line Gy14 were milder, and only slight chlorosis developed (Figure 1A4–A6). The accumulation of H_2_O_2_ after staining with DAB solution could be detected as brown spots in both lines from 1 dpi (Figure 1B). In the Gy14 line the H_2_O_2_ accumulation was more abundant in comparison to the susceptible B10 line, when the size and color intensity of precipitates were taken as indicators of H_2_O_2_ production (Figure 1B4–B6 vs. B1–B3, Supplementary Figure S1). The level of H_2_O_2_ accumulation increased between 1 and 3 dpi in both lines as the area occupied by precipitates enlarged substantially (Figure 1B2,B5 vs. B3,B6). The strong outbreak of H_2_O_2_ production in resistant Gy14 suggests the important role of H_2_O_2_ in response to pathogen attack. The microscopic examinations of samples collected from edges of infection lesions (Figure 1C) indicated that bacteria with well-preserved protoplasts were present in intercellular spaces and inside degraded mesophyll cells in the B10 line (Figure 1C2,C3), whereas they were found only in intercellular spaces in the Gy14 line (Figure 1C5,C6). Although no macroscopically visible symptoms of infection appeared on leaves of both lines at 1 dpi (Figure 1A2,A5), bacteria were found frequently in the B10 leaf mesophyll at 1 dpi (Figure 1C2) and only occasionally in leaves of Gy14 plants (Figure 1C5). At 3 dpi bacteria formed extensive and numerous colonies in B10 leaves, and they appeared less numerous in Gy14 leaves (Figure 1C3,C6). No indications of bacteria deterioration were observed. Mesophyll cells being in contact with bacteria were strongly deteriorated in both genotypes at 1 dpi (Figure 1C2,C5). Their cytoplasm collapsed, and together with deteriorating nuclei, mitochondria and chloroplasts they formed masses of strongly osmiophilic remnants.

Chloroplast envelopes and the internal system of thylakoids and grana were destroyed; thus, chloroplasts could be recognized only due to the presence of electron-translucent starch grains (Figure 1C2,C5). At 3 dpi in B10 leaves, colonies of bacteria were surrounded by mesophyll cells with completely degraded cytoplasm containing destroyed mitochondria, nuclei and chloroplasts (Figure 1C3). In contrast, at the same time point, in Gy14 plants bacteria were less numerous, and they were still present only in intercellular spaces (Figure 1C6). Mesophyll cells surrounding intercellular spaces with bacteria were plasmolyzed, but their cytoplasm remained well preserved and electron-translucent similarly to control leaves (Figure 1C6 vs. C4). Vacuole, mitochondria, chloroplasts and nuclei were still clearly recognizable (Figure 1C6). In some chloroplasts, features indicating their deterioration occurred, such as swelling of the thylakoid lumen and change of their outlines from crescent-like into round. In general, it can be speculated that in Gy14 plants mesophyll cells react with protoplast collapse at initial stages of interaction (1 dpi), but thereafter mesophyll cells at some distance from the infection point become plasmolyzed but remain alive at 3 dpi, thus resisting pathogen attack and restricting the spread of infection and development of lesions.

### 2.2. Transcriptome Profiling

**Figure 2:**
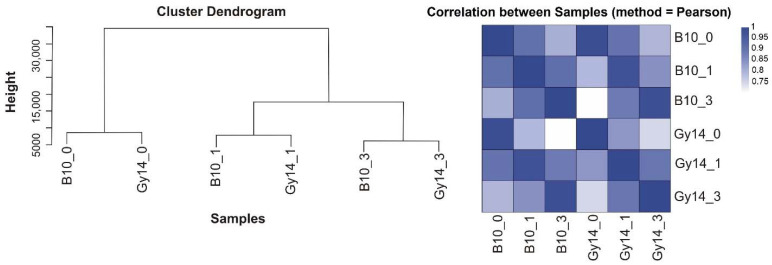
Hierarchical clustering and heatmap of Pearson’s correlation (R^2^) between B10 and Gy14 lines inoculated with *Psl* 814/98 strain at 0, 1 and 3 dpi. (**A**) Hierarchical clustering between samples; (**B**) correlation matrix of expression profiles, both X-axis and Y-axis represent each sample. Correlated profiles are colored from blue to white (high to low correlation). Abbreviations: B10_0–B10 line at 0 dpi (control), B10_1–B10 line at 1 dpi, B10_3–B10 line at 3 dpi, Gy14_0–Gy14 line at 0 dpi (control), Gy14_1–Gy14 line at 1 dpi, Gy14_3–Gy14 line at 3 dpi.

The early response of susceptible cucumber line B10 and resistant Gy14 to *Psl* 814/98 infection was examined using RNA-seq at 0, 1 and 3 dpi. In total about 44.7 Mb of clean reads was obtained per each sample. Reads were mapped on cucumber reference genome 9930 v. 2 at a similar ratio, on average 93.59%. A slightly higher number of expressed genes was found in the susceptible B10 line than in the resistant Gy14 line at all time points. There were 18,859 (0 dpi), 19,039 (1 dpi) and 19,149 (3 dpi) transcripts detected for B10 and 18,807 (0 dpi), 18,871 (1 dpi) and 18,981 (3 dpi) transcripts detected for Gy14. On average 40% of clean reads were mapped uniquely to the reference transcripts in all samples (Supplementary Table S1). Gene expression levels were calculated for each sample, and both hierarchical clustering and Pearson’s correlation of transcriptomic profiles showed the tremendous effect of inoculation on cucumber gene expression. The transcription profiles at 0, 1 and 3 dpi were similar irrespective of the cucumber line, although the expression profiles for 3 dpi samples were more distant than for 1 dpi ones as compared to control (0 dpi) (Figure 2).

### 2.3. Differentially Expressed Genes

**Figure 3:**
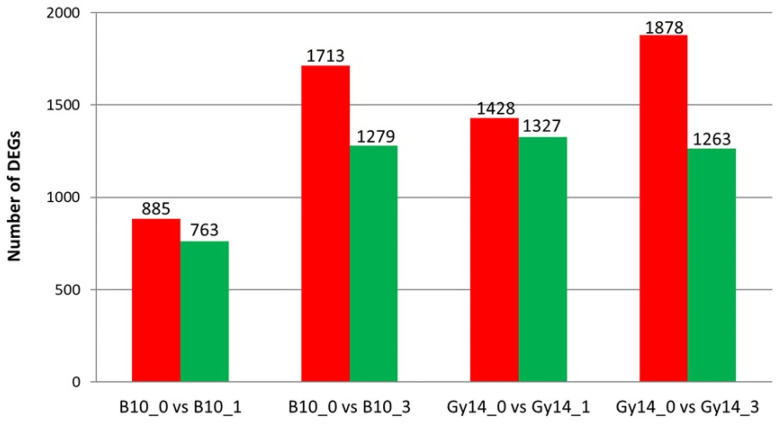
Summary of the differentially expressed genes (DEGs) identified for Gy14 and B10 lines at 1 and 3 dpi in comparison to 0 dpi. Numbers indicate DEGs detected in each pairwise comparison. Red color represents up-regulated genes, whereas green color corresponds to down-regulated genes. Abbreviations: B10_0–B10 line at 0 dpi (control), B10_1–B10 line at 1 dpi, B10_3–B10 line at 3 dpi, Gy14_0–Gy14 line at 0 dpi (control), Gy14_1–Gy14 line at 1 dpi, Gy14_3–Gy14 line at 3 dpi.

Based on gene expression profiling, differentially expressed genes (DEGs) were detected for each line (log2 fold-change ≥2, false discovery rate FDR ≤ 0.001) (Supplementary Figure S2, Supplementary Table S2). The results confirmed the distinct response of B10 and Gy14 to *Psl* 814/98 infection. There were 1648 DEGs detected for the susceptible B10 line at 1dpi (885 up- and 763 down-regulated) and 2992 DEGs at 3 dpi (1713 up- and 1279 down-regulated). In contrary, there were 2755 DEGs detected for the resistant Gy14 line at 1 dpi (1428 up- and 1327 down-regulated) and 3141 DEGs at 3 dpi (1878 up- and 1263 down-regulated) (Figure 3). Although more DEGs were identified in both lines at 3 dpi, the number of up- and down-regulated DEGs at 1 dpi was higher in the resistant Gy14 than in susceptible B10 line, which suggests that the dynamic transcriptomic response plays a key role in cucumber resistance to *Psl*.

**Figure 4:**
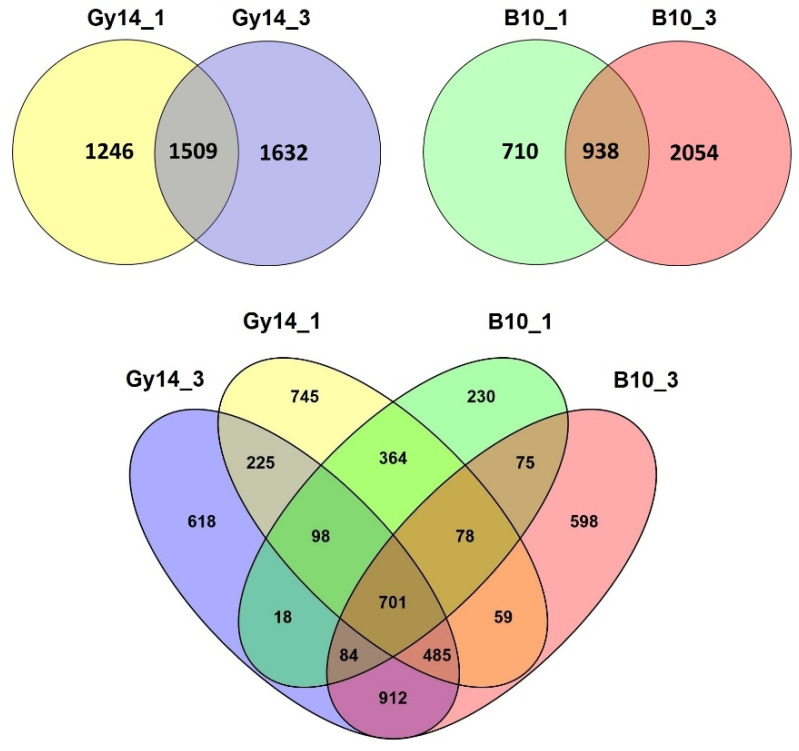
Venn diagrams representing the distribution of differentially expressed genes (DEGs) in the resistant Gy14 line and susceptible B10 line at 1 and 3 dpi with *Psl* 814/98. Abbreviations: B10_1–B10 line at 1 dpi, B10_3–B10 line at 3 dpi, Gy14_1–Gy14 line at 1 dpi, Gy14_3–Gy14 line at 3 dpi.

The total number of DEGs identified at both time points in the Gy14 line was 1509 (34% of all DEGs), whereas in line B10 it was 938 (25% of all DEGs). Moreover, there were as many as 1246 DEGs expressed at 1 dpi in resistant Gy14 line, whereas there were only 710 unique DEGs at 1 dpi in the susceptible B10 line. The more detailed analysis of DEGs revealed that 701 DEGs were common in both lines at both time points. Additionally, 364 DEGs were in common for both lines at 1 dpi and 912 DEGs at 3 dpi. On the other hand, there were 745 genes at 1 dpi and 618 at 3 dpi uniquely expressed in the Gy14 line, whereas only 230 genes were uniquely expressed at 1 dpi and 598 at 3 dpi in the B10 line (Figure 4).

**Figure 5:**
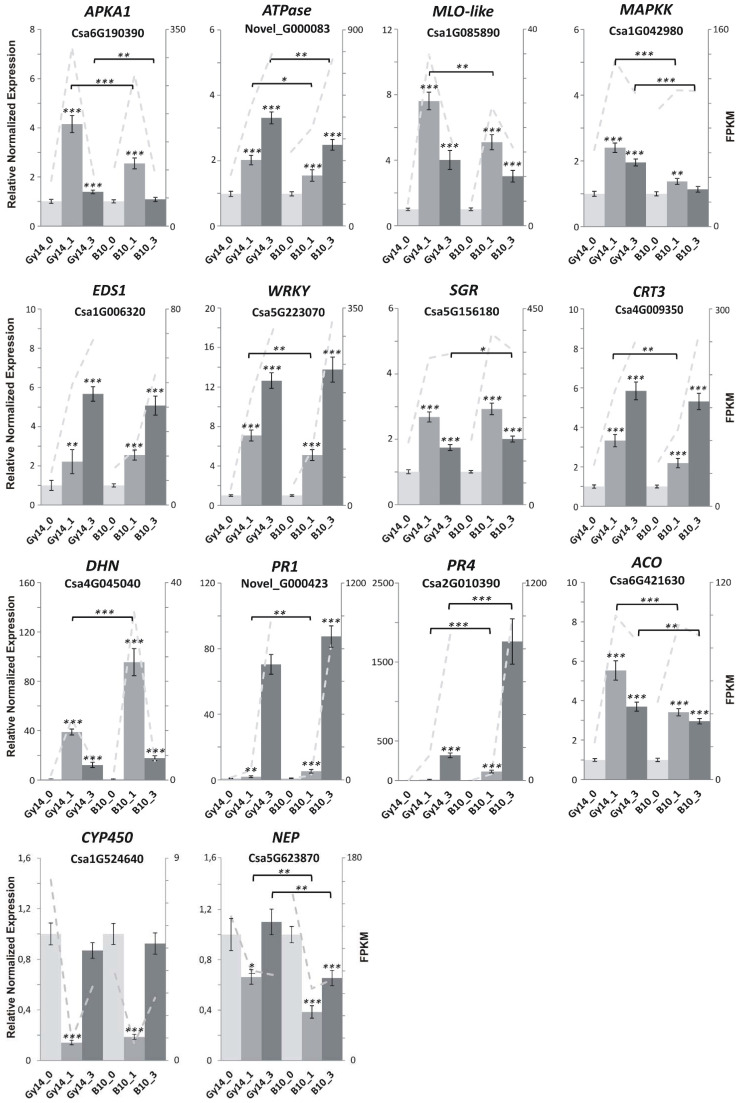
The expression of 14 selected differentially expressed genes (DEGs) for lines Gy14 and B10 at 0, 1 and 3 dpi. Diagrams show relative normalized gene expression level ±SEM revealed by RT-qPCR from 3 biological and 3 technical replicates. For data normalization, 2 reference genes *CACS* and *TIP41* were used. Grey dashed line represents estimated numbers of fragments per kilobase million (FPKM). Full gene names are provided in Supplementary Table S3. Significance levels were calculated with Student’s *t*-test: *p* < 0.05 (*), *p* < 0.01 (**) and *p* < 0.001 (***). Abbreviations: B10_0–B10 line at 0 dpi (control), B10_1–B10 line at 1 dpi, B10_3–B10 line at 3 dpi, Gy14_0–Gy14 line at 0 dpi (control), Gy14_1–Gy14 line at 1 dpi, Gy14_3–Gy14 line at 3 dpi.

Subsequently, 14 *Psl*-regulated genes were used for validation of RNA-seq results by RT-qPCR (Supplementary Table S3). The gene expression levels obtained in both analyses, RT-qPCR and RNA-seq, were positively correlated, although the gene expression level estimated by numbers of fragments per kilobase million (FPKM) was usually higher as compared to normalized relative expression revealed by RT-qPCR (Figure 5).

### 2.4. Functional Categories of DEGs

The classification of genes involved in cucumber response to *Psl* 814/98 was performed using Gene Ontology (GO) analysis. DEGs were classified into main GO categories: biological process, cellular component and molecular function (Supplementary Figure S3). In terms of GO biological process the highest number of DEGs was assigned to three categories: metabolic process, cellular process and single-organism process. There were also DEGs assigned to the following categories: localization, response to stimulus, biological regulation and others. In terms of GO cellular component, the majority of DEGs were assigned to the categories cell and cell part, membrane and membrane part as well as organelle and organelle part. In terms of GO molecular function, most genes were assigned to two categories: catalytic activity and binding. For major GO categories, a similar number of genes was assigned to each category except for B10 line at 1 dpi (Supplementary Figure S3).

**Figure 6:**
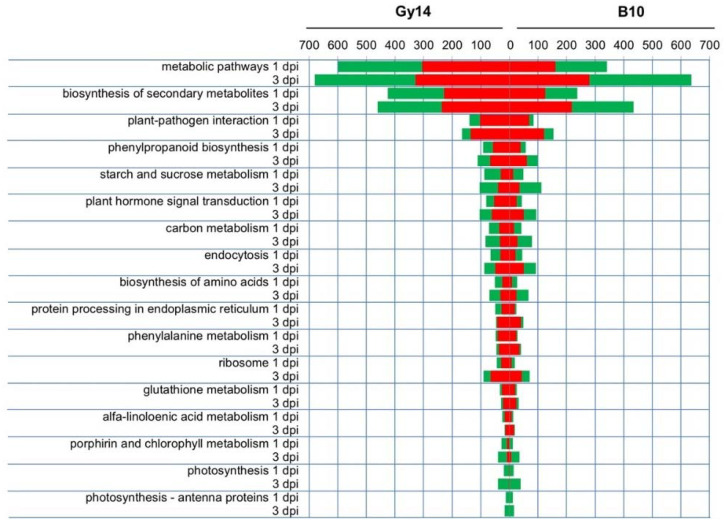
Summary of the Kyoto Encyclopedia of Genes and Genomes (KEGG) pathway enrichment analysis in Gy14 and B10 lines at 1 and 3 dpi. Red and green indicate up- and down-regulated differentially expressed genes (DEGs), respectively.

Analysis of significantly enriched pathways according to the Kyoto Encyclopedia of Genes and Genomes (KEGG) revealed that the highest numbers of genes in both lines and time points were related to metabolic pathways, biosynthesis of secondary metabolites and plant–pathogen interaction. Other enriched KEGG pathways were phenylpropanoid biosynthesis, plant hormone signal transduction, carbon metabolism, endocytosis, starch and sucrose metabolism. Similarly to the GO analysis, the number of identified DEGs was the lowest in the B10 line at 1 dpi. Genes classified to the KEGG pathways involving phenylalanine metabolism, glutathione metabolism, α-linolenic acid metabolism, ribosome and protein processing in the endoplasmic reticulum were up-regulated in both lines and time points. In processes of porphyrin and chlorophyll metabolism as well as photosynthesis and photosynthesis-antenna, nearly all expressed genes in both lines and time points were down-regulated (Figure 6).

**Figure 7:**
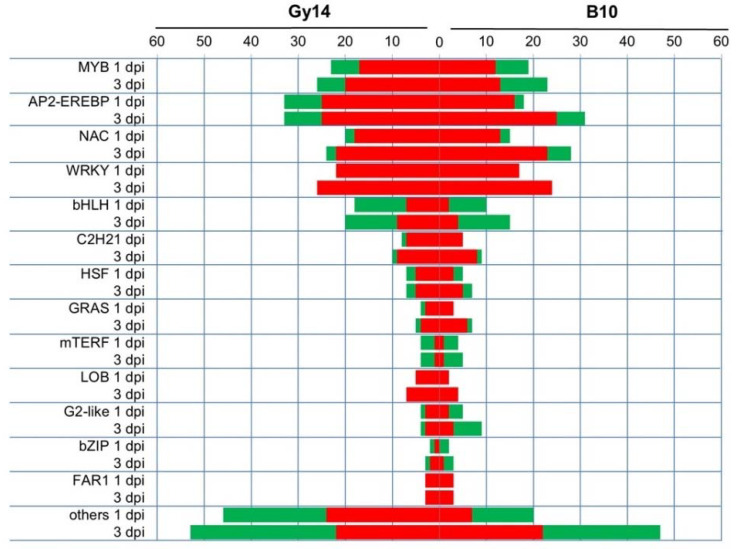
The summary of differentially expressed transcription factors (TF-DEGs) in Gy14 and B10 lines at 1 and 3 dpi. Names of transcription factor families/subfamilies are based on classification implemented by the Plant Transcription Factor Database [31]. Red and green indicate up- and down-regulated TF-DEGs, respectively.

Transcriptional regulation of genes plays a crucial role in plant defense to pathogens. In this study several groups of differentially regulated transcription factors (TF-DEGs) were identified (Figure 7, Supplementary Table S6). In total 198 TF-DEGs (131 up- and 67 down-regulated) and 225 TF-DEGs (158 up- and 67 down-regulated) were identified in the Gy14 line at 1 and 3 dpi, respectively, as well as 128 TF-DEGs (86 up- and 42 down-regulated) and 215 TF-DEGs (142 up- and 73 down-regulated) in the B10 line at 1 and 3 dpi as compared to 0 dpi, respectively (Figure 7, Supplementary Figure S4B). Among them, 56 TF-DEGs were commonly found in each line and time point. In contrary, 52 and 48 TF-DEGs were unique for Gy14 at 1 and 3 dpi, whereas only 18 and 38 TF-DEGs were unique for B10 at 1 and 3 dpi, respectively (Supplementary Figure S4A). Identified TF-DEGs belonged to several families, with the highest number of TF-DEGS from AP2-EREBP, MYB, NAC, bHLH and WRKY families (Figure 7). The highest number of up-regulated TF-DEGs in the Gy14 line belonged to AP2-EREBP, WRKY, NAC and MYB families, and the highest number of down-regulated TF-DEGs belonged to bHLH, AP2-EREBP and MYB families. The lowest overall number of TF-DEGs was detected in the B10 line at 1 dpi. At 3 dpi the number of up-regulated TF-DEGs from AP2-EREBP, WRKY and NAC groups strongly increased, whereas the number of down-regulated TF-DEGs belonging to bHLH, MYB and G2-like families decreased. Interestingly, in the case of TF-DEGs belonging to the WRKY family, no down-regulated genes, neither in the Gy14 nor B10 line, at any timepoint were detected. Identified DEGs encoding different groups of transcription factors reveal the complexity of transcriptional regulation of genes involved in cucumber response to *Psl*.

### 2.5. Genes Related to Defense Response

Numerous genes involved in defense response of cucumber to *Psl* 814/98 were identified (Supplementary Table S2). Among them, there were genes expressed only in resistant or susceptible lines as well as common for both lines. Several genes encoding homologs of leucine-rich repeat (LRR) family proteins were found. Some of them were up-regulated only in the Gy14 line at 1 dpi (e.g., Csa6G425100) or in Gy14 at 1 and 3 dpi and in B10 line at 3 dpi (e.g., Csa6G425100). Others (e.g., Csa2G404910 and Csa7G284420) were up-regulated in both lines at 1 and 3 dpi. Putative homolog of LRR receptor-like serine/threonine-protein kinase Flagellin Sensing 2 (FLS2) (Csa6G522700), involved in the perception of bacterial flagellin, was up-regulated in both lines at 1 and 3 dpi. We found genes encoding putative receptor-like kinases/receptor-like proteins (RLKs/RLPs) that were up-regulated only in Gy14 (Csa3G651750 and Csa3G730960) or in both lines (Csa4G289120 and Csa4G290150).

Cucumber homolog of Enhanced Disease Susceptibility 1 (EDS1) (Csa1G006320), a key regulator of the immune response in plants, which takes part in salicylic acid (SA) signaling, was up-regulated in both lines (Figure 5). In contrast, homolog of Nonexpressor of Pathogenesis-related Genes 1 (NPR1) (Csa4G063470), which also is involved in SA signaling, was highly up-regulated only in Gy14 at 1 dpi. Several DEGs related to jasmonic acid (JA) biosynthesis and signaling showed expression changes in both lines. Genes encoding lipoxygenases (LOX) (Csa4G286960, Csa4G288610 and Csa7G449420) were up-regulated at both time points and lines, whereas genes encoding allene oxide cyclase 1 (AOC1) (Csa5G366670) and Jasmonate ZIM-domain proteins (JAZ) (Csa1G597690 and Csa3G645940) were up-regulated in Gy14 at 1 and 3 dpi, but in B10 they were up-regulated only at 3 dpi. Genes encoding proteins involved in ethylene biosynthesis and signaling, such as aminocyclopropane-1-carboxylate synthase (ACS) (Csa6G006800 and Csa4G049610), were up-regulated in both lines at 1 and 3 dpi, whereas the gene encoding ethylene-insensitive protein 3 (EIN3) (Csa6G051520) was down-regulated in both lines and time points (Supplementary Table S2).

Genes encoding proteins involved in defense response were highly up-regulated in response to *Psl* 814/98 infection in both lines, e.g., phenylalanine ammonia-lyase (PAL) (Novel_G000584, Csa4G008250, Csa4G008260, Csa4G008770, Csa4G008760, Csa6G446280, Csa6G446290 and Csa6G445780) (Supplementary Table S2). Similarly, genes encoding pathogenesis-related (PR) proteins, i.e., PR1 (Novel_G000423), PR2 (Csa1G616240, Csa1G660200 and Csa1G555080), PR3 (Csa6G507520 and Csa6G509040) and PR4 (Csa2G010390) were highly up-regulated in both lines at 3 dpi. The gene encoding defense protein 19kDa dehydrin (DHN) (Csa4G045040) was significantly up-regulated in both lines at 1 dpi (Figure 5, Supplementary Table S2). In conclusion, distinct transcriptomic response of both cucumber lines (B10 and Gy14) to *Psl* 814/98 infection was identified.

## 3. Discussion

Bacterial ALS disease remains one of the most destructive cucumber maladies. Reports from the previous years informed about serious ALS outbreaks, for example in Iran and China [32,33]. Moreover, ALS results in damage of the leaves that can lead to secondary *P. cubensis* infection and even higher yield reductions [34]. Additionally, Newberry et al. [35,36] reported that novel pathotypes of *P. syringae* complex can cause disease symptoms on multiple cucurbit hosts (watermelon, cantaloupe and squash). The most virulent strains of *P. syringae* pv. *lachrymans* belong to phylogroup 3 based on multi-locus sequence typing (MLST) [7], which refer to genomospecies 2 according to Gardan et al. [37]. The most severe symptoms on cucumber leaves become visible seven days after infection [13,38]. In this study we have investigated the early response of cucumber to the highly virulent *Psl* strain 814/98 belonging to phylogroup 3 and genomospecies 2. Cucumber line Gy14, which is an important source of resistance, and susceptible inbred line B10 were studied at the 1st and the 3rd day post inoculation at cytological, morphological and transcriptomic levels.

The changes in infected leaves were evaluated at macro- and microscopic levels. It was observed that *Psl* 814/98 infects both lines, although bacteria expand and reproduce more effectively in susceptible line B10. This could be seen well, especially in microscopic observations where bacteria were present in intercellular spaces and inside deteriorated cells. In the B10 line, bacteria were observed in the leaf mesophyll from 1 dpi, and numerous bacteria masses were observed at 3 dpi (Figure 1). In contrary, in the Gy14 line bacteria in small amounts were observed only in intercellular spaces of leaf tissue, and mesophyll cells next to them were plasmolyzed, but remained alive at 3 dpi (Figure 1C4–C6 vs. C1–C3). These microscopic observations were reflected by macroscopic observations—symptoms of infection appeared as angular water-soaked lesions and chlorosis, particularly severely developed on leaves of the susceptible B10 plants (Figure 1A1–A6). In contrast, the accumulation of H_2_O_2_, detected as brown spots in DAB-stained leaves, was stronger in resistant line Gy14, indicating the important role of reactive oxygen species (ROS) in managing early defense response of cucumber to *Psl* (Figure 1B1–B6). Similar results were shown in other studies with different cucumber pathogens. Wang et al. [22] showed that in the resistant line of cucumber infected with *C. cassiicola,* greater H_2_O_2_ accumulation was observed at 1 dpi in resistant than in susceptible line. In addition, Xu et al. [39] pointed out that after powdery mildew inoculation, DAB precipitations increased at 2 dpi in resistant cucumber SSL508-28 as compared to susceptible D8, indicating higher H_2_O_2_ accumulation in the resistant line. The accumulation of ROS might trigger expression of genes coding for oxidative metabolism and ROS scavenging enzymes such as peroxidases, catalases, thioredoxins as well as non-enzymatic antioxidant biosynthesis (i.e., ascorbic acid, glutathione, flavonoids and carotenoids) [40]. In our study we noted that the genes encoding ROS scavenging enzymes and glutathione *S*-transferases were, overall, up-regulated except for thioredoxins genes (Supplementary Table S2).

We observed that chlorosis was developed after inoculation in leaves of both lines, although it was more progressive in the susceptible B10 line. We detected down-regulation of several genes related to chlorophyll metabolism and photosynthesis (Figure 6), which in conjunction with degradation of chloroplasts (Figure 1C) confirms that chloroplast-related metabolism is important in response to pathogen attack. Wang et al. [14] proposed the *CsSGR* gene (Csa5G156180), a key regulator in the chlorophyll degradation pathway, as a candidate gene of triple resistance to ALS, downy mildew and anthracnose. We found that expression of *CsSGR* significantly increased at 1 dpi and decreased at 3 dpi in both lines, remaining significantly higher at 3 dpi in susceptible B10 (Figure 5, Supplementary Table S2), which is overall in accordance with results of Wang et al. [14], where the elevated expression of *CsSGR* in the susceptible line 9930 after inoculation with *P. cubensis* was observed.

Bacterial attack on the plant cell sets off a complex of defense reactions to combat pathogens. The receptors located on the cell membrane recognize pathogen effectors, activate signaling routes, resulting in activation of the network of transcription factors that control genes encoding plant defense proteins. These are part of pathogen-associated molecular pattern (PAMP) triggered immunity (PTI) and effector-triggered immunity (ETI) that follow each other or occur in parallel after pathogen attack [41,42]. In PTI, evolutionary conserved PAMPs such as flagellins, chitins or lipopolysaccharides are detected by plant receptors at the plasma membrane and are responsible for pathogen recognition. These events entail multiple responses and activation of complex signaling cascades, which involve mitogen-activated and calcium-dependent protein kinases or reactive oxygen species [43]. In this study, transcriptomic analysis revealed a massive defense response at the early stage of *Psl* 814/98 infection in the resistant Gy14 line. The number of DEGs was higher in Gy14 (2755 DEGs) compared to the susceptible line B10 (1648 DEGs) at 1 dpi (Figure 3). Similar response of the resistant line was reported in studies focused on the cucumber response to oomycete pathogen *P. cubensis,* where at 1 dpi the number of DEGs was higher in the resistant PI197088 line (4864 DEGs) than in susceptible Vlaspik (1969 DEGs) [20]. In contrary, dynamic transcriptomic response was shown at 2 dpi in the susceptible Beijing204 line comparing to resistant line D1322 upon infection with fungal pathogen *A. cucumerina* [25]. Our results suggest that massive transcriptomic response is pivotal in cucumber defense to *Psl* strain 814/98 similarly to the response to oomycete and fungal pathogens.

In this study we found several genes encoding putative receptors. Cucumber homolog of *FLS2* encoding bacterial flagellin receptor was up-regulated in both lines (Supplementary Table S2). A cucumber homolog of *EDS1* (Csa1G006320), which is a central regulatory point interconnecting different mechanisms of plant immunity, was expressed in a similar manner in both lines (Figure 5, Supplementary Table S2). In contradiction, cucumber *EDS1* was down-regulated after fungus *S. fuliginea* infection [24]. We found also numerous genes encoding transcription factors that were differentially expressed in response to *Psl* inoculation. A much higher number of TF-DEGs was identified in the resistant Gy14 line than in susceptible B10 (Figure 7 and Supplementary Figure S4). Interestingly, all identified TF-DEGs belonging to the WRKY group were up-regulated. For example, the Csa5G223070 gene, encoding WRKY transcription factor, was up-regulated in both lines and time points, with a significantly higher expression level at 1 dpi in the resistant Gy14 line (Figure 5, Supplementary Table S2). Yang et al. [44] reported that three WRKY genes, including Csa5G223070, were highly induced in resistant line No.26 after *B. cinerea* infection. Expression of this WRKY gene increased also during defense responses against powdery mildew [24] and *P. melonis* infections [21]. Our study also suggests the possible role of Csa5G223070 in building up a defense response to pathogens.

As a result of ETI and/or PTI, the final step of defense response is transcriptional induction of genes encoding proteins involved in production of various defense metabolites and pathogenesis-related proteins (PR) [41,43]. In our study several genes encoding phenylalanine ammonia-lyases (PALs), key enzymes involved in phenylpropanoid metabolism, and several PRs and other defense-related proteins were found to be differentially expressed in both lines. In cucumber, 13 *PAL* genes were identified, and multiple tandem duplications of those genes were shown [45,46]. In our study eight *PAL* genes highly up-regulated in both lines (Novel_G000584, Csa4G008250, Csa4G008260, Csa4G008770, Csa4G008760, Csa6G446280, Csa6G446290, Csa6G445780) were detected (Supplementary Table S2). These genes represent major *PAL* blocks located at chromosomes 4 and 6. Similarly to our study, in cucumber infected with the CCYV virus, 11 differentially expressed *PAL* genes were detected [17].

Extreme changes in expression levels were revealed for genes encoding different classes of PR proteins (i.e., Novel_G000423, Csa1G616240, Csa1G660200, Csa1G555080, Csa6G507520, Csa6G509040 and Csa2G010390). In general, after nearly lack of expression of these genes at 0 dpi, their expression increased at 1 dpi and became yet higher at 3 dpi (Supplementary Table S2). Expression of cucumber *PR1* (Novel_G000423, not annotated in 9930 v. 2 genome corresponding to CsGy7G006240 in Gy14 v. 2), encoding a protein with homology to *A. thaliana* cysteine-rich secretory protein PR1 (AT3G04720), was at a significantly higher level in susceptible B10 at 1 dpi and reached the highest level of expression in both lines at 3 dpi (Figure 5). Expression of cucumber *PR4* (Csa2G010390), encoding a protein homologous to *A. thaliana* PR4 (AT3G04720), increased in both lines at 1 and 3 dpi with a significantly higher level in susceptible line B10 (Figure 5). There were relatively lower expressions of *PR1* and *PR4* genes in resistant line Gy14. In the study performed by Wang et al. [22], strong expression of the *PR4* gene was observed in response to fungus *C. cassiicola* in both resistant and susceptible lines. Other authors also pointed out activation of this gene in cucumber response to various pathogens [16,23,47]. Our study shows that the expression of cucumber *PR4* gene (Csa2G010390) is possibly important in building a response to *Psl* 814/98. Interestingly, we found that in both lines gene encoding defense protein 19kDa dehydrin (Csa4G045040), corresponding to *CsDHN2* described by Zhou et al. [48], was significantly up-regulated. Dehydrins belong to the late embryogenesis abundant (LEA) protein family, which are multifunctional proteins involved in response to different stresses [49]. *CsDHN2* was highly activated at 1 dpi in both lines with significantly higher expression level in susceptible line B10, but at 3 dpi expression dropped to the level observed in resistant Gy14 line (Figure 5). Promoters of cucumber *PR4* and *CsDHN2* could be useful in developing an experimental model to study, in detail, the molecular mechanism of cucumber response to *P. syringae* pv. *lachrymans* infection.

## 4. Materials and Methods

### 4.1. Plant Materials and Inoculation

Seeds of 2 cucumber (*Cucumis sativus* L.) inbred lines, B10 and Gy14, were planted into Jiffy-7 peat pellets (Jiffy International AS, Kristiansand, Norway). Line Gy14 is characterized as resistant to ALS, and line B10 is susceptible to ALS [6,38]. Well-characterized in our previous studies, the virulent *Psl* 814/98 strain was used for plant inoculation [7,50,51]. Plants were grown in a Conviron CMP6050 growth chamber (Conviron, Winnipeg, Canada) at 25 °C day and 22 °C night, at 16 h photoperiod under fluorescent white light (300 μmol m^−2^ s^−1^) and 65% relative humidity. Plants were inoculated at the 2nd to 3rd leaf stage by spraying the abaxial side of each leaf with inoculum concentration of 1 × 10^7^ CFU mL^−1^ prepared and using methodology as described by Olczak-Woltman et al. [38]. Inoculated plants were kept in darkness for 24 h at 22 °C, under relative humidity close to 100%, that was lowered to 90% for successive days. The aerial part of plants (i.e., leaves, cotyledons and hypocotyls) were collected from six randomly selected plants in each of three biological replicates and for each time point, i.e., before inoculation (0 dpi), one day post inoculation (1 dpi) and three days post inoculation (3 dpi) with *Psl* strain 814/98. One biological replicate was used for sequencing and three replicates for RT-qPCR validation.

### 4.2. Histochemical Staining

To detect H_2_O_2_ in the inoculated cucumber plants, DAB staining was performed [52]. Leaves of cucumber B10 and Gy14 lines, collected at 0, 1 and 3 dpi, were soaked in DAB solution (Sigma, St. Louis, MO, USA) and incubated on a rotary shaker at 80× rpm for 4–5 h. After incubation, the leaves were placed in glass jars in DAB solution and vacuum-treated for 15 min to improve DAB infiltration. Later, leaves were transferred to the distaining solution (ethyl alcohol: glacial acetic acid: glycerol, 3:1:1, *v*/*v*/*v*) and heated until the tissues became transparent. Images obtained after DAB staining were quantified using ImageJ software [53]. Each sample was represented by four replicates.

### 4.3. Microscopic Examination of Leaf Tissue

Fragments of cucumber leaf blades (2 × 2 mm in size) were dissected with sharp blades from at least 5 different leaves of B10 and Gy14 lines at 0, 1 and 3 dpi. They were immediately transferred and immersed in a fixative composed of 2% (*w*/*v*) paraformaldehyde and 2% (*v*/*v*) glutaraldehyde in 0.1 M sodium cacodylate buffer (pH 7.2). After 1 h the fixative was replaced with a new one, and samples were left in open vials at room temperature under -0.4 MPa vacuum for 2 h. Afterwards, samples were washed 4 times in the same buffer for 10 min each and dehydrated in a 10% (*v*/*v*) graded ascending series of aqueous mixtures of ethanol for 30 min each. The pure ethanol was substituted by propylene oxide, and samples were infiltrated with ascending mixtures of epoxy resin with the propylene oxide for 24 h [54]. Resin-infiltrated samples were transferred into flat embedding molds filled with pure epoxy resin. The resin was polymerized at 65 °C for 16 h. For transmission electron microscopy examinations, 90 nm thick sections were taken with a Leica UCT ultramicrotome (Leica, Wetzlar, Germany) and collected on copper grids. They were contrasted with methanolic solution of uranyl acetate and aqueous solution of lead nitrate. Grids were examined under an FEI M268D ‘Morgagni’ transmission electron microscope (FEI Corp., Hillsboro, OR, USA) operating at 80 kV and equipped with an SIS ‘Morada’ digital camera (SIS, Münster, Germany) operating at 10 MPix resolution. Images were cropped, resized and equalized for similar contrast and brightness using Adobe Software Package (Adobe, Santa Clara, CA, USA).

### 4.4. RNA Isolation, cDNA Library Construction and RNA-seq

Total RNA was isolated using RNeasy Plant Mini Kit followed by DNase I treatment using an RNase-Free DNase I Set (Qiagen, Hilden, Germany). The quality and concentration of RNA was monitored using agarose gel electrophoresis and a NanoDrop2000 spectrophotometer (Thermo Fisher Scientific, Waltham, MA, USA). Libraries were synthesized, and sequencing was performed at BGI-Tech (Shenzhen, China). Six barcoded cDNA libraries from pooled samples representing three biological replicates were prepared. Agilent 2100 Bioanalyzer (Agilent Technologies, Santa Clara, CA, USA) and ABI StepOnePlus Real-Time PCR System (Thermo Fisher Scientific) were used for quality check and quantification of the libraries. cDNA libraries were multiplexed and sequenced as a single run using the Illumina HiSeq4000 system (Illumina, San Diego, CA, USA). Sequenced data were deposited as BioProject PRJNA704621 at the National Center for Biotechnology Information (NCBI, Bethesda, MD, USA).

### 4.5. Identification of Differentially Expressed Genes (DEGs)

Raw sequence reads were filtered, and low-quality reads, adaptor-polluted or with a high content of unknown bases were removed. Clean reads were mapped to the cucumber reference genome 9930 v. 2 (http://cucurbitgenomics.org/, accessed on 1 March 2021) [55]. Transcript reconstruction was performed using StringTie v. 1.0.4 [56]. Cuffcompare v. 2.2.1 of Cufflinks program [57] was used to compare reconstructed transcripts to the reference, and an additional 592 novel coding transcripts were predicted (Supplementary Table S7). Clean reads were mapped to the transcripts using Bowtie2 v. 2.2.5 [58], and then the gene expression level was calculated with RSEM v. 1.2.12 providing estimate data of the fragments per kilobase of transcript per million mapped reads (FPKM) for each transcript [59]. Pearson correlation and hierarchical clustering between samples were performed for FPKM using cor and hclust functions in R.

NOIseq and Poisson distribution method [60] were used for identification of differentially expressed genes presented as matrix fold-change of expression profiles (FPKM) for each treated sample (FPKM_Gy14_1, FPKM_Gy14_3, FPKM_B10_1 and FPKM_B10_3) compared to untreated sample (FPKM_Gy14_0 and FPKM_B10_0). DEGs were calculated as log2 fold-change (FPKM_treated sample/ FPKM_untreated sample). According to the Poisson distribution methodology, corrected false discovery rate (FDR) ≤ 0.001 and log2 fold-change ≥ ±2.0 were set as the threshold for significant differential expression. The clustering analysis of the expressed genes between each sample were shown in a Venn diagram using Venny 2.1 [61].

### 4.6. Functional Classification of DEGs

The Gene Ontology (GO) classification [62], including three ontologies—biological process, cellular component and molecular function—as well as Kyoto Encyclopedia of Genes and Genomes (KEGG) classification and pathways analyses of the DEGs were performed [63]. The phyper function in R was used, and *p*-values ≤ 0.05 were considered as significant. Getorf from the EMBOSS package v. 6.5.7.0 [64] and hmmsearch v. 3.0 [65] were used to predict ORF for each DEG, then ORFs were aligned to TF domains in the PlnTFDB database, and differentially expressed genes encoding transcription factors (TF-DEGs) were annotated according to PlnTFDB [31].

### 4.7. RT-qPCR Analysis

The concentration and the quality of RNA was verified after DNase I treatment using NanoDrop 2000 Spectrophotometer. The concentration of purified RNA was adjusted to 100 ng μL^−1^. cDNA was synthesized using a Transcriptor High-Fidelity cDNA Synthesis Kit (Roche, Basel, Switzerland) in a 20 μL reaction tubes using oligo(dT) primers according to the manufacturer’s instructions. The expression study was carried out using a CFX96 Touch cycler (Bio-Rad Laboratories, Hercules, CA, USA) with Master Mix Maxima SybrGreen qPCR MM 2×ROX (Thermo Fisher Scientific) according to the manufacturer’s instructions. For qPCR, 4 μL of cDNA was used. The qPCR program was performed as follows: 50 °C for 2 min to activate Maxima DNA polymerase, 95 °C for 10 min, followed by 39 thermal cycles of 15 s at 95 °C and 1 min at 58 °C for DEGs or at 60 °C for reference genes. Melting curve analysis was performed immediately after the qPCR. The temperature range used for the melting curve generation was between 70 and 95 °C. All RT-qPCR assays were performed in 3 biological and 3 technical replicates for each tested line as well as for each tested time point, in the presence of 2 negative controls (sterile water as a template).

### 4.8. Validation of RNA-seq Results

To validate the expression level of differentially expressed genes, estimated in RNA-seq analysis real-time reverse transcription PCR (RT-qPCR) was performed. Primers were designed for selected DEGs using Primer3 [66] and checked for complementarity with cucumber genome sequences of lines Gy14 (http://cucurbitgenomics.org/, accessed on 1 March 2021) and B10 [67]. Primer names and sequences are listed in Supplementary Table S3. Reference genes for data normalization in RT-qPCR were selected as described earlier [68]. Ten candidate reference genes (Supplementary Table S4) were analyzed using geNorm v. 3.4 [69], NormFinder v. 0.953 [70] and BestKeeper v. 1.0 [71]. The results generated by each applet were used to calculate the geometric mean (GeoMean) in the RefFinder program [72]. Two genes encoding clathrin adaptor complex subunit (CACS) and TIP41-like family protein (TIP41) were ranked as the most stable (Supplementary Table S5) and used as reference genes. The expression levels of the DEGs were calculated using the 2^−ΔΔC^_T_ method [73] and CFX Manager v. 3.1 (Bio-Rad Laboratories).

## 5. Conclusions

This study provides transcriptome data for cucumber infected with the bacterial pathogen *P. syringae* pv. *lachrymans* and reveals the complexity of cucumber transcriptomic response to this pathogen. Both cytological and transcriptomic evaluations indicate that dynamic response to the invading pathogen plays an important role in host resistance. Numbers of genes involved in host-specific response to the highly virulent *Psl* 814/98 strain were detected. The differences in expression between resistant and susceptible cucumber lines were revealed, although many genes encoding proteins involved in the defense response were differentially expressed in both lines. Our results suggest that massive transcriptomic response is pivotal in the cucumber defense to bacterial pathogens.

## Data Availability

Sequenced data were deposited as BioProject PRJNA704621 at the National Center for Biotechnology Information (NCBI, Bethesda, MD, USA).

## Supplementary Materials

The following are available online at https://www.mdpi.com/article/10.3390/ijms22084192/s1, Figure S1. Percentage of DAB staining sites quantified by ImageJ software; Figure S2. Volcano plots of cucumber genes expressed in plants of resistant Gy14 and susceptible B10 lines at 1 and 3 dpi compared to 0 dpi; Figure S3. Gene Ontology (GO) classification of DEGs from Gy14 and B10 lines at 1 and 3 dpi; Figure S4. Differentially expressed genes encoding transcription factors (TF-DEGs) identified in B10 and Gy14 lines at 1 and 3 days after inoculation with *P. syringae* pv. *lachrymans* strain 814/98; Table S1. Summary of the RNA sequencing (RNA-seq) and mapping of the reads for two cucumber lines Gy14 and B10 at 0, 1 and 3 dpi with *P. syringae* pv. *lachrymans* strain 814/98; Table S2. Detailed lists of differentially expressed genes (DEGs) detected in Gy14 and B10 lines at 1 and 3 dpi with *P. syringae* pv. *lachrymans* strain 814/98; Table S3. List of the genes and primer sequences used for validation of differentially expressed genes (DEGs) by RT-qPCR; Table S4. Primer sequences and description of candidate reference genes for RT-qPCR to study gene expression in cucumber inoculated with *P. syringae* pv. *lachrymans* strain 814/98; Table S5. Overall ranking of candidate RT-qPCR reference genes analyzed by geNorm, NormFinder, BestKeeper and RefFinder applets; Table S6. Detailed lists of differentially regulated transcription factors (TF-DEGs) detected in Gy14 and B10 lines at 1 and 3 dpi with *P. syringae* pv. *lachrymans* strain 814/98; Table S7. List of novel transcripts.
