# Supplementary material for: Transcriptome Profiling of Cucumber (Cucumis sativus L.) Early Response to Pseudomonas syringae pv. lachrymans
Source: Int J Mol Sci. 2021 Apr 18;22(8):4192. doi: 10.3390/ijms22084192 (PMC8072787; doi:10.3390/ijms22084192)
Supplement: Supplementary file 1 [file ijms-22-04192-s001.zip › Suppl_tableS1.pdf]

Supplementary Table S1. Summary of the RNA sequencing (RNA-seq) and mapping of the reads for two cucumber lines Gy14 and B10 at 0, 1 and 3 days post inoculation with *P. syringae* pv. *lachrymans* strain 814/98. As a reference cucumber genome 9930 v.2 was used. Each sample represents pool of three biological replicates.

Sample	Total raw reads (Mb)	Total clean reads (Mb)	Clean reads Q30 (%)	Reference genome mapping		Summary of gene expression		
				Total mapping ratio (%)	Uniquely mapping ratio (%)	Total transcript number	Total mapping ratio (%)	Uniquely mapping ratio (%)
Gy14_0 dpi	48.11	44.91	97.00	93.70	92.23	18,807	75.10	41.09
Gy14_1 dpi	48.11	44.57	96.69	93.47	91.97	18,871	76.21	40.23
Gy14_3 dpi	48.11	44.63	96.65	93.18	91.64	18,981	73.53	39.74
B10_0 dpi	48.11	44.81	96.88	93.75	92.27	18,859	74.53	40.26
B10_1 dpi	48.11	44.77	97.00	93.74	92.27	19,039	75.15	39.45
B10_3 dpi	48.11	44.56	96.91	93.69	92.18	19,149	73.12	39.42
