# Supplementary material for: Transcriptome Profiling of Cucumber (Cucumis sativus L.) Early Response to Pseudomonas syringae pv. lachrymans
Source: Int J Mol Sci. 2021 Apr 18;22(8):4192. doi: 10.3390/ijms22084192 (PMC8072787; doi:10.3390/ijms22084192)
Supplement: Supplementary file 1 [file ijms-22-04192-s001.zip › Suppl_tableS3.pdf]

Supplementary Table S3. List of the genes and primer sequences used for validation of differentially expressed genes (DEGs) by RT-qPCR.

No.	Abbreviation	Cucumber gene ID 9930 v.2	Gene description 9930 v.2	Cucumber gene ID Gy14 v.2	Gene description Gy14 v.2	*A. thaliana* ortholog gene ID	*A. thaliana* gene description TAIR10	Primer sequences (5' → 3') forward/reverse
1	*ACO*	Csa6G421630	1-aminocyclopropane-1-carboxylate oxidase	CsGy6G021870	1-aminocyclopropane-1-carboxylate oxidase 1	AT1G05010	Ethylene-forming enzyme	CGAATCAAACATCTCCCAGA<br>CCTTTTCCAACCCAAGATTC
2	*APKA1*	Csa6G190390	Probable receptor-like protein kinase	CsGy6G014300	Protein kinase APK1A	AT1G07570	Protein kinase superfamily protein APKA1	TGGGTTTTCCAAAGACACAG<br>ATGGTCGATTTTTGTCAACG
3	*ATPase*	Novel_G000083	-	CsGy2G006330	Cation-transporting P-type ATPase	AT2G18960	$H^{(+)}$-ATPase 1	CAGGATTTCGTTGGTATCGT<br>TGATGCATCTTGTTCACACC
4	*CRT3*	Csa4G009350	Calreticulin-3	CsGy4G002380	Calreticulin-3	AT1G08450	Calreticulin 3	AGGGGCAGAATTATCCCATA<br>CAGGATGTCCCAATCACTGT
5	*CYP450*	Csa1G524640	Cytochrome P450	CsGy1G022780	Cytochrome P450	AT2G29090	Cytochrome P450	CGGAGATCAACAAGATTTGG<br>CACCTCCATGCTCTTTGTTC
6	*DHN*	Csa4G045040	Dehydrin	CsGy4G005210	Dehydrin Xero 1	AT5G66400	Dehydrin family protein	TAAGGAGCATGGTGGCATAC<br>TTGACACTGCTTGCTCTTCA
7	*EDS1*	Csa1G006320	Enhanced disease susceptibility 1 protein	CsGy1G001860	Protein EDS1L-like	AT3G48090	Alpha/beta-Hydrolases superfamily protein	GGAAGCTTAAGGAGGTTCTGA<br>TCTGGGTTTCTTTGCTGTTC
8	*MAPKK*	Csa1G042980	Mitogen-activated protein kinase kinase	CsGy1G007240	Mitogen-activated protein kinase kinase 9	AT1G73500	MAP kinase kinase 9	TAAACCGTCGAATCTGTTGG<br>CGTAAGAATTGCAAGCATCC
9	*MLO-like*	Csa1G085890	MLO-like protein	CsGy1G012830	MLO-like protein	AT1G61560	Seven transmembrane MLO family protein	CACGTTGAGATGGGAGGTTA<br>GGGCCTCCAATATGAATCTC
10	*NEP*	Csa5G623870	Aspartic proteinase nepenthesin-1	CsGy5G027100	Eukaryotic aspartyl protease family protein	AT3G54400	Eukaryotic aspartyl protease family protein	TCGTCGATATTCCTCCCTCT<br>TGTTACGTTTCTCCCCACTC
11	*PR1*	Novel_G000423	-	CsGy7G006240	Cysteine-rich venom protein	AT2G14610	Pathogenesis-related gene 1	ACCTGTCAGTTGGGATGAAA<br>GCACGAGTTTGAGGCATAAT
12	*PR4*	Csa2G010390	Wound-induced protein WIN1	-	-	AT3G04720	Pathogenesis-related 4	ATACGGTTGGACTGCCTTCT<br>AAATCTAGCCCTCCATTTGC
13	*SGR*	Csa5G156180	Senescence-inducible chloroplast stay-green protein	CsGy5G003280	STAYGREEN protein	AT4G22920	Non-yellowing 1	TTCTCTCTTCCCCTGTAAACG<br>GGAAATTTGCCTGGATGTTT
14	*WRKY*	Csa5G223070	WRKY transcription factor	CsGy5G008920	WRKY transcription factor 34	AT2G40750	WRKY DNA-binding protein 54	TGTCCTGATCATTCCCTTTG<br>CTTTTTGGCCGTACTTCCTC
