# Supplementary material for: Transcriptome Profiling of Cucumber (Cucumis sativus L.) Early Response to Pseudomonas syringae pv. lachrymans
Source: Int J Mol Sci. 2021 Apr 18;22(8):4192. doi: 10.3390/ijms22084192 (PMC8072787; doi:10.3390/ijms22084192)
Supplement: Supplementary file 1 [file ijms-22-04192-s001.zip › Suppl_tableS4.pdf]

Supplementary Table S4. Primer sequences and description of candidate reference genes for RT-qPCR to study gene expression in cucumber inoculated with *Pseudomonas syringae* pv. *lachrymans* strain 814/98.

No.	Abbr.	NCBI GeneBank accession number	Gene description	Primer sequences (5' → 3') forward/reverse	Reference
1	*ACT*	AB010922	*Actin*	CCGTTCTGTCCCTCTACGCTAGTG	Migocka and Papierniak (2010)
				GGAACTGCTCTTTGCACTCTCGAG	
2	*CACS*	GW881874	*Clathrin adaptor complex subunit*	TGGGAAGATTCTTATGAAGTGC	Migocka and Papierniak (2010)
				CTCGTCAAATTTACACATTGGT	
3	*F-box*	GW881870	*F-box protein/ galactose oxidase*	GGTTCATCTGGTGGTCTT	Migocka and Papierniak (2010)
				CTTTAAACGAACGGTCAGTCC	
4	*HEL*	GW881869	*Helicase*	TTCTCGAAGATTTAGTGATTCATGTG	Migocka and Papierniak (2010)
				CAATGGACGAATGCAAAGG	
5	*TIP41*	GW881871	*TIP41-like family protein*	CAACAGGTGATATTGGATTATGATTATAC	Migocka and Papierniak (2010)
				GCCAGCTCATCCTCATATAAG	
6	*TUA*	AJ715498	*α - Tubulin*	ACGCTGTTGGTGGTGGTAC	Wan et al. (2010)
				GAGAGGGGTAAACAGTGAATC	
7	*TUB*	–	*α - Tubulin*	CCTCGACATTGAGCGACCTAAC	Kowalczuk et al. (2014)
				CATCCACGTTCAATGCACCA	
8	*UBI-1*	AF104391	*Ubiquitin-like protein*	CCTTATTGACCAACCAGTAGT	Migocka and Papierniak (2010)
				GGACAATGTTGATTTCCTCG	
9	*UBI-ep*	AY372537	*Ubiquitin extension protein*	CACCAAGCCCAAGAAGATC	Wan et al. (2010)
				TAAACCTAATCACCACCAGC	
10	*YSL8*	GW881872	*Mitosis protein*	CCTTGTGGATATCACAGAAGTT	Migocka and Papierniak (2010)
				CTTGTTTATCCTTGAGTGCC	

References

Kowalczuk C, Pawełkowicz M, Chądzyńska K, Paziewska A, Wóycicki R, Ostrowski J, Cebula J, Przybecki Z. Evaluation of reference genes transcription stability in generative whorls of cucumber flower (*Cucumis sativus* L.). 2014. In: Biotechnology and Plant Breeding Perspectives, Behl RK, Arseniuk E (eds.), Agrobios International, Jodhpur, India, pp.275-286.

Migocka M, Papierniak A. Identification of suitable reference genes for studying gene expression in cucumber plants subjected to abiotic stress and growth regulators. 2010. Mol. Breed. 28:343-357.

Wan H, Zhao Z, Qian C, Sui Y, Malik AA, Chen J. Selection of appropriate reference genes for gene expression studies by quantitative real-time polymerase chain reaction in cucumber. 2010. Anal. Biochem. 399:257-261.
