# Supplementary material for: Transcriptome Profiling of Cucumber (Cucumis sativus L.) Early Response to Pseudomonas syringae pv. lachrymans
Source: Int J Mol Sci. 2021 Apr 18;22(8):4192. doi: 10.3390/ijms22084192 (PMC8072787; doi:10.3390/ijms22084192)
Supplement: Supplementary file 1 [file ijms-22-04192-s001.zip › Suppl_tableS5.pdf]

Supplementary Table S5. Overall ranking of candidate RT-qPCR reference genes analyzed by geNorm, NormFinder, BestKeeper and RefFinder applets.

Rank (weight)	Applet						Overall ranking RefFinder	
	geNorm v.3.4		NormFinder v.0.953		BestKeeper v.1.0			
	Gene	*M-value*	*Gene*	*Stability value*	*Gene*	*Stability value*	*Gene*	*GeoMean*
1	*TUA*	0.094	*CACS*	0.261	*TIP41*	0.265	*CACS*	1.86
2	*TUB*	0.094	*ACT*	0.292	*UBI-ep*	0.298	*TIP41*	2.82
3	*ACT*	0.362	*TIP41*	0.428	*CACS*	0.329	*ACT*	3.03
4	*CACS*	0.456	*HEL*	0.434	*UBI-1*	0.399	*TUB*	4.28
5	*F-BOX*	0.524	*UBI-1*	0.471	*HEL*	0.449	*TUA*	4.41
6	*HEL*	0.557	*TUA*	0.514	*F-BOX*	0.454	*HEL*	4.68
7	*TIP41*	0.585	*TUB*	0.514	*ACT*	0.513	*UBI-1*	5.32
8	*UBI-1*	0.610	*UBI-ep*	0.544	*TUB*	0.586	*UBI-ep*	5.83
9	*UBI-ep*	0.623	*F-BOX*	0.550	*TUA*	0.588	*F-BOX*	7.02
10	*YSL8*	0.708	*YSL8*	0.947	*YSL8*	0.903	*YSL8*	10.00
