# Supplementary figures and images for: Transcriptome Profiling of Cucumber (Cucumis sativus L.) Early Response to Pseudomonas syringae pv. lachrymans
Source: Int J Mol Sci. 2021 Apr 18;22(8):4192. doi: 10.3390/ijms22084192 (PMC8072787; doi:10.3390/ijms22084192)

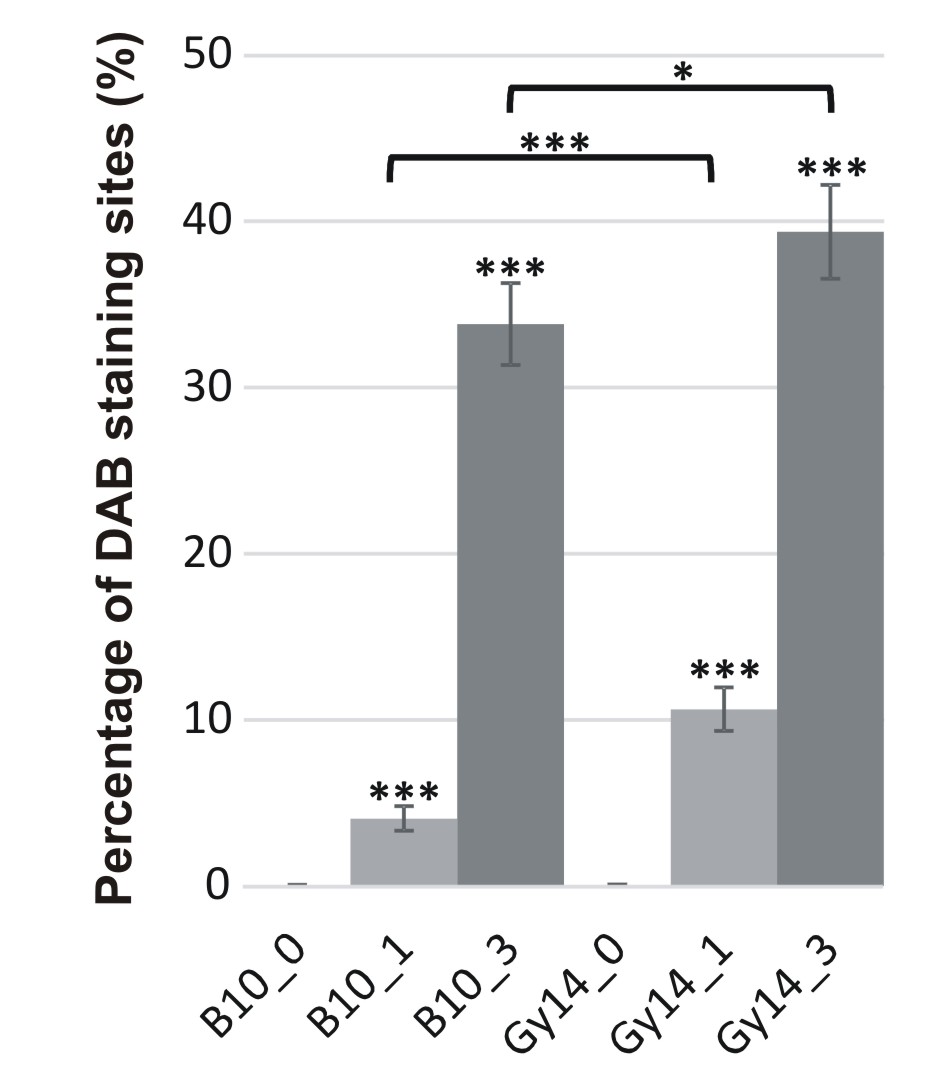

Supplement: Supplementary file 1 [file ijms-22-04192-s001.zip › Supplementary Figure S1.jpg]

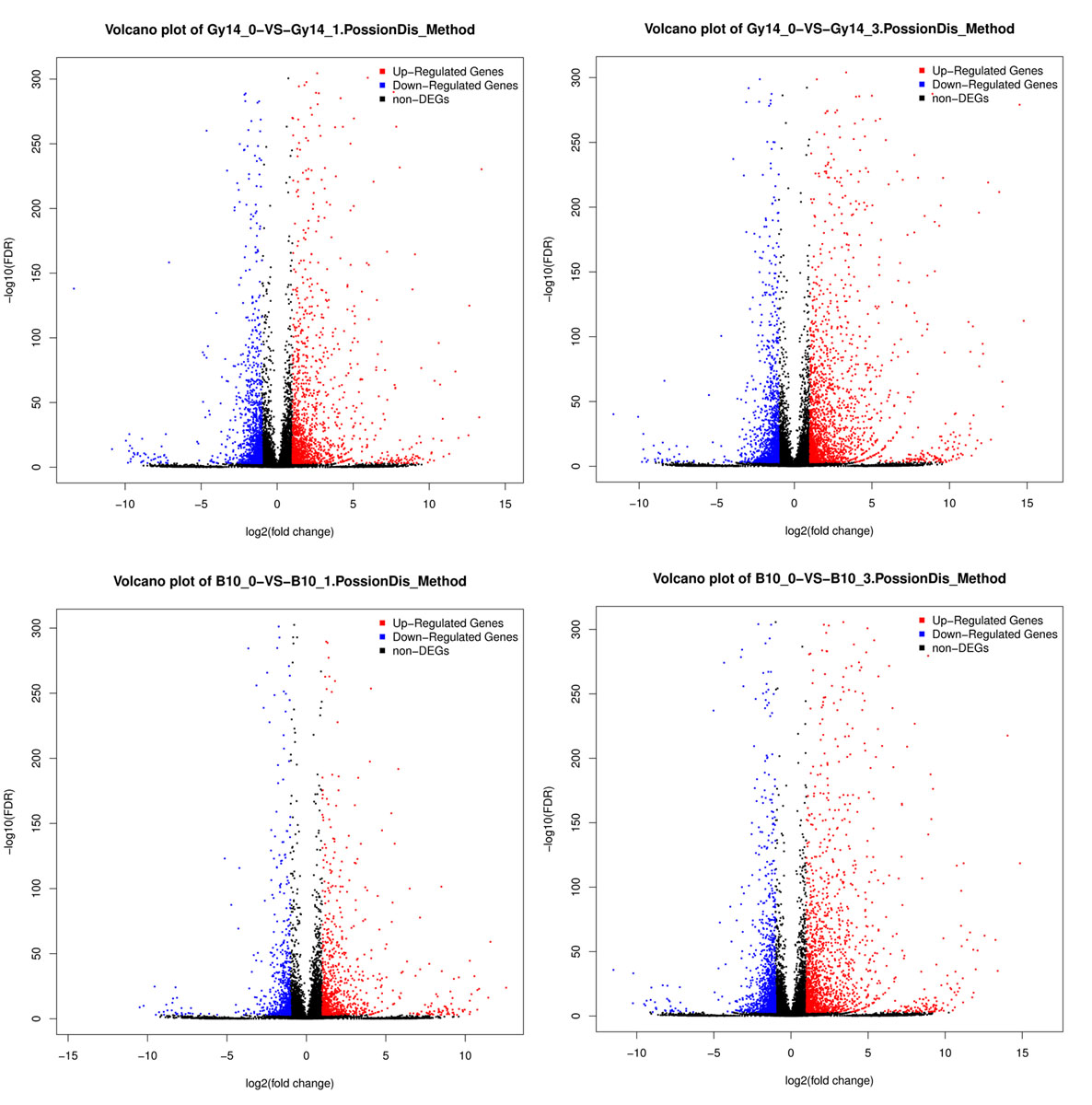

Supplement: Supplementary file 1 [file ijms-22-04192-s001.zip › Supplementary Figure S2.jpg]

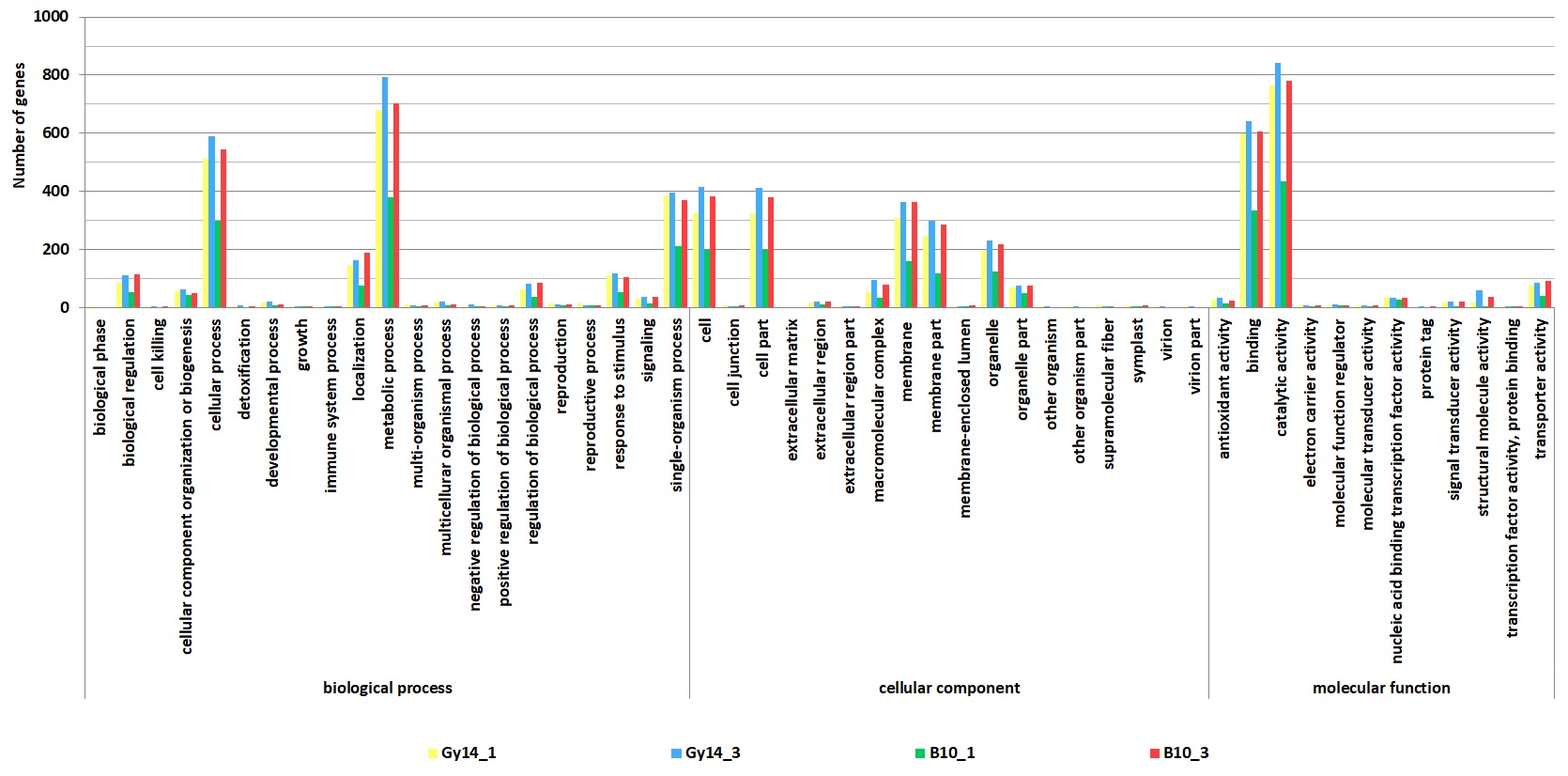

Supplement: Supplementary file 1 [file ijms-22-04192-s001.zip › Supplementary Figure S3.jpg]

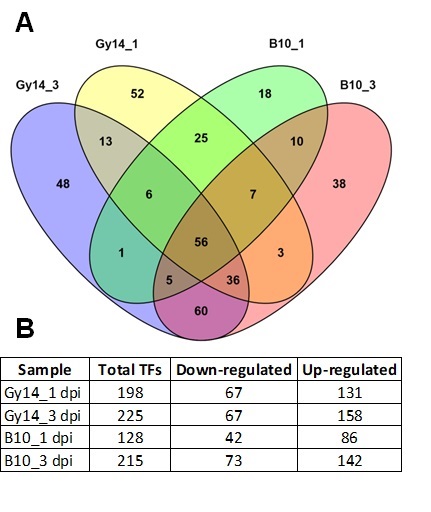

Supplement: Supplementary file 1 [file ijms-22-04192-s001.zip › Supplementary Figure S4.jpg]
